# Discovery of unobservable parameters via physical embedding

**DOI:** 10.1093/nsr/nwag508

**Published:** 2026-08-19

**Authors:** Le Cheng, Xiaoran Liu, Lingjin Kong, Haitao Zhao, Jun Xiong, Fanglin Gu, Xiaoying Zhang, Baoquan Ren, Jibo Wei, Hao Yin

**Affiliations:** College of Electronic Science and Technology, National University of Defense Technology, Changsha 410073, China; College of Electronic Science and Technology, National University of Defense Technology, Changsha 410073, China; College of Electronic Science and Technology, National University of Defense Technology, Changsha 410073, China; College of Electronic Science and Technology, National University of Defense Technology, Changsha 410073, China; College of Electronic Science and Technology, National University of Defense Technology, Changsha 410073, China; College of Electronic Science and Technology, National University of Defense Technology, Changsha 410073, China; College of Electronic Science and Technology, National University of Defense Technology, Changsha 410073, China; Academy of Military Science, Beijing 100076, China; College of Electronic Science and Technology, National University of Defense Technology, Changsha 410073, China; College of Electronic Science and Technology, National University of Defense Technology, Changsha 410073, China; Academy of Military Science, Beijing 100076, China

**Keywords:** inverse problems, non-identifiability, differentiable physics, wireless communications, MRI

## Abstract

Recovering a source signal from indirect measurements often depends on parameters that cannot be observed directly, such as wireless channel states or MRI coil sensitivities. We introduce Physics-Embedded Inverse Learning (PEIL), which embeds a fixed, differentiable inverse solver and uses signal recovery to supervise their estimation without parameter labels. PEIL makes parameter selection task-optimal: each setting is judged by the reconstruction it produces through the fixed solver, rather than by its agreement with nominal labels. In locally non-identifiable regimes, different settings yield nearly equivalent reconstructions, allowing the loss to favour those better suited to the fixed solver. Non-identifiability thus becomes a resource for recovery. In high-mobility wireless communications, PEIL generalises to unseen channel profiles and velocities without retraining and reaches comparable recovery performance using 20-fold fewer training samples than baselines trained with parameter labels. At high SNR, it also achieves a lower symbol error rate than an oracle-reference least-squares baseline using true pilot-channel values and fixed interpolation. In parallel MRI, without sensitivity-map labels or a separate calibration step, PEIL discovers stable coil sensitivity maps with magnitude structure consistent with a calibration reference and reconstructs anatomically faithful images. Together, these results show that a fixed inverse solver can make unobservable parameters learnable from signal recovery.

## Teaser

Signal recovery supervises unobservable parameter estimation without parameter labels through a fixed, differentiable inverse solver embedded in learning, as demonstrated in wireless communications and MRI.

## INTRODUCTION

Many scientific and engineering systems seek to recover a source signal from indirect, noisy measurements. In high-mobility wireless communications, a receiver must extract transmitted symbols despite unknown time-varying multipath channels and carrier-frequency offsets [1]. In magnetic resonance imaging (MRI), an anatomical image must be reconstructed from undersampled frequency-domain data acquired through coils with unknown sensitivity profiles [2,3]. Despite their different physical mechanisms, these problems share a common inverse structure (Fig. 1a). A forward operator $\mathcal {F}(\mathbf {X}; \boldsymbol{\theta }, \mathbf {H})$ couples the source signal $\mathbf {X}$ with system parameters $\boldsymbol{\theta }$, such as carrier-frequency offset, and high-dimensional latent parameters $\mathbf {H}$, such as channel states or coil sensitivity maps. Because recovery depends on quantities that are not observed directly, reconstructing $\mathbf {X}$ from $\mathbf {Y}$ is generally ill-posed [4–6].

**Figure 1.**
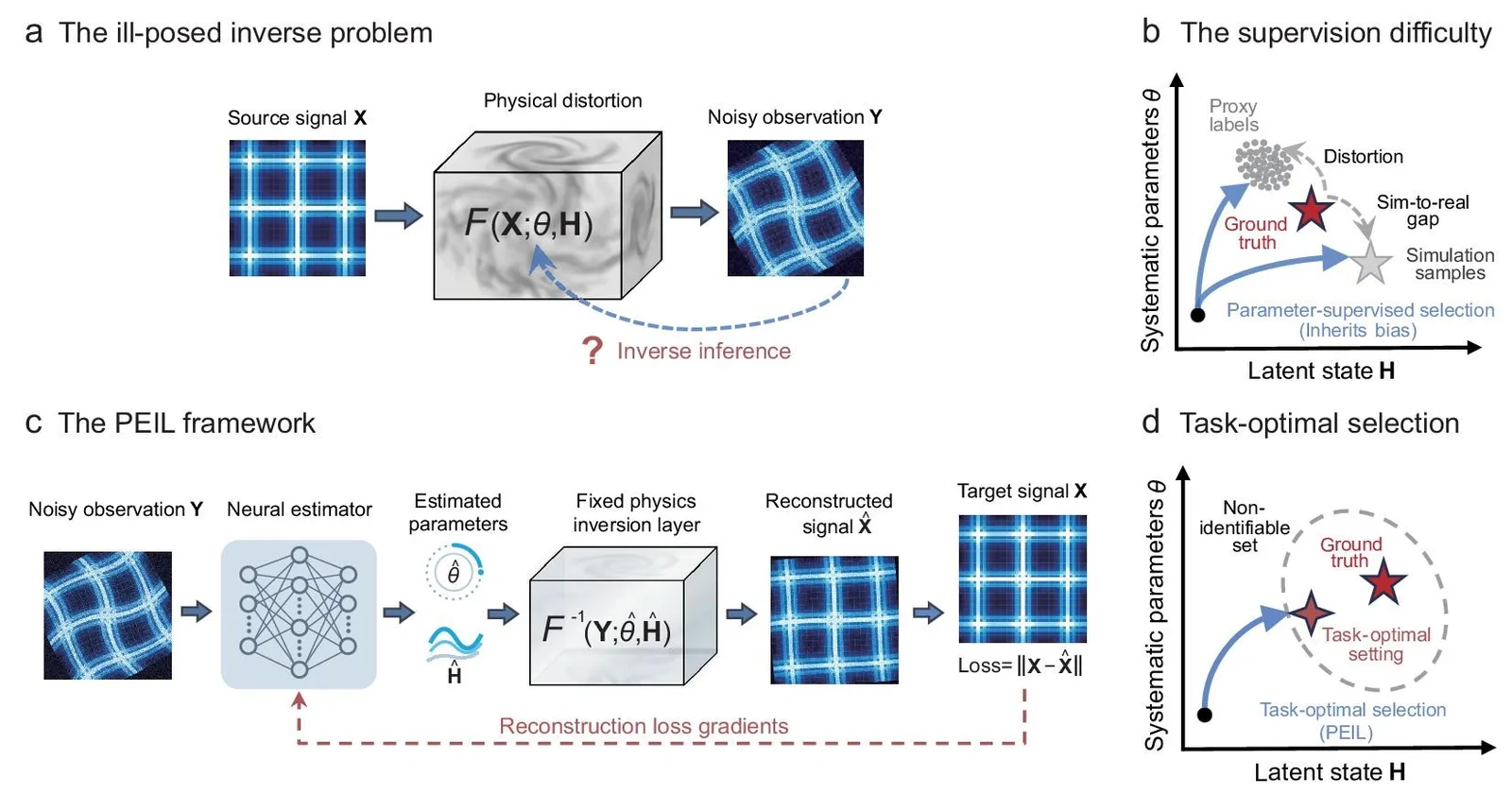
Problem structure and the PEIL framework. (a) The ill-posed inverse problem. A forward operator $\mathcal {F}(\mathbf {X}; \boldsymbol{\theta }, \mathbf {H})$ couples the source signal $\mathbf {X}$ with system parameters $\boldsymbol{\theta }$ and high-dimensional latent parameters $\mathbf {H}$ to produce observations $\mathbf {Y}$. Only $\mathbf {Y}$ is observed, and it is indirect, noisy, and partially informative. The quantities required to recover $\mathbf {X}$ are not observed directly. (b) The supervision difficulty. Because these parameters are not directly labelled, parameter-supervised training must rely on proxies from classical estimation or calibration (grey point cloud), or on simulated samples with a reality gap (grey star). (c) PEIL. A neural estimator predicts solver-conditioning quantities from $\mathbf {Y}$. A fixed, differentiable inverse solver with no learnable parameters then uses them to reconstruct $\hat{\mathbf {X}}$. Reconstruction gradients pass through the solver to train the estimator without parameter labels. (d) Non-identifiability and task-optimal parameter selection. Different solver-conditioning settings can yield nearly equivalent reconstructions, forming a local non-identifiable set at the reconstruction level (dashed circle). PEIL is trained through signal recovery and can select a task-optimal setting within this set.

The standard response is explicit calibration: pilot-based estimation in wireless systems or sensitivity estimation in MRI. Calibration supplies approximate values for the hidden parameters, but consumes measurement resources or adds estimation steps. Direct neural models avoid explicit parameter estimation by mapping measurements to the target signal [7,8]. However, they discard the known inverse physics, often require large training sets, and can generalise poorly outside the training distribution [9–12]. A more constrained route is to retain the fixed inverse solver and learn only the quantities it requires. The obstacle is supervision. Instantaneous channel state information (CSI) is not directly observable in wireless systems [13], and exact coil sensitivities lack independent references in MRI [14]. Their labels must therefore come from classical estimation or from simulations with a reality gap [10] (Fig. 1b). Can signal recovery itself supervise these unobservable parameters?

Here we introduce Physics-Embedded Inverse Learning (PEIL). PEIL embeds the known inverse physics as a fixed, differentiable solver and learns only the quantities required to condition it. A neural estimator infers these quantities, including carrier-frequency offset (CFO), CSI, and coil sensitivities, from the measurement $\mathbf {Y}$. The fixed solver then uses them to reconstruct $\!\!\hat{\mathbf {\,\,\,X}}$ from $\mathbf {Y}$ (Fig. 1c). Because the solver is differentiable [15–17], the reconstruction loss backpropagates through the physical inversion into the estimator. Physical embedding thus turns signal recovery into supervision for parameters that cannot be labelled directly.

Under PEIL, a parameter setting is termed task-optimal when it is selected by downstream reconstruction performance through the fixed solver, rather than by proximity to a nominal label. Different settings can leave the recovered signal essentially unchanged [18,19]. In orthogonal frequency-division multiplexing (OFDM), for example, a residual CFO deviation can be absorbed by a corresponding phase rotation in the channel response. In parallel MRI, coil sensitivities and the underlying image share a scale–phase ambiguity. Settings connected by such ambiguities form a local non-identifiable set at the reconstruction level. The nominal setting is only one representative, and reconstruction loss supplies the criterion for choosing among them. With an exact solver, this choice would not affect recovery. With a practical fixed solver, interpolation and modelling errors can make some representatives better suited than the nominal one. Local non-identifiability can therefore become a resource when reconstruction loss favours coordinated deviations that improve the recovered signal.

PEIL belongs to the broader paradigm of differentiable inverse learning, alongside deep unrolled and bilevel methods [20–22]. Its defining constraint is that the solver algorithm, update rule, and solver-side parameters remain fixed and non-learnable. The estimator can affect reconstruction only through explicit conditioning quantities; there is no learned post-inversion module to correct the result. Conditioning errors that harm recovery therefore remain visible to the reconstruction loss. By contrast, many unrolled and bilevel methods use learned updates or regularisers [23–27]; physics-informed networks typically impose physical laws as soft constraints [28–30]; and analysis-by-synthesis methods optimise observation agreement without an explicit inverse path [31]. In PEIL, the fixed solver provides the common reconstruction pathway linking the predicted conditioning quantities to signal recovery. Domain-specific auxiliary losses may additionally constrain an ill-posed decomposition, as in MRI.

We evaluate PEIL on two inverse problems governed by different physical mechanisms. The primary testbed is a high-mobility OFDM communication system [32], where channel parameters are tightly coupled and rapidly time-varying. PEIL generalises across unseen channel conditions without retraining and reaches comparable recovery performance with 20-fold fewer training samples than baselines trained with parameter labels. It also achieves a lower symbol error rate (SER) at high signal-to-noise ratio (SNR) than an oracle-reference least-squares (LS) baseline supplied with the exact CFO and true pilot-channel values under the same fixed interpolation. To test the same principle in another inverse problem, we apply PEIL to parallel MRI [14], where coil sensitivities and the underlying image are coupled. Without sensitivity-map labels or a separate calibration step, PEIL discovers stable coil sensitivity maps with magnitude structure consistent with a calibration reference and reconstructs anatomically faithful images.

## RESULTS

### Parameter accuracy and signal recovery in high-mobility wireless channels

We first evaluate PEIL in a high-mobility wireless system with 3GPP Extended Vehicular A (EVA) fading [33], where transmitted symbols are multiplicatively coupled with CSI and CFO-induced phase. The scalar system parameter *θ* is the residual CFO induced by oscillator mismatch and bulk Doppler shift. The latent parameter $\mathbf {H}$ is the instantaneous CSI. Neither is directly observable at the receiver. This setting tests whether signal recovery can supervise these unobservable quantities and whether their nominal values are necessarily the best inputs to a fixed receiver.

To separate parameter accuracy from recovery performance, all implemented receiver methods use the same fixed Gaussian interpolation operator to obtain dense CSI. Oracle-ref. LS [34] uses the exact CFO and supplies this operator with ground-truth channel values at the pilot positions. Its residual dense-channel error therefore arises from the fixed interpolation step. Supervised AGM [35] is a state-of-the-art estimator trained with explicit parameter labels. Supervised Solver shares PEIL’s estimator backbone but trains its output heads directly against CFO and CSI labels; PEIL instead trains through the fixed receiver using the final symbol reconstruction loss. The analytical Oracle Bound [36] is reported separately as a reference under perfect parameter knowledge.

Without CFO or CSI labels, PEIL recovers physically meaningful estimates. Its scalar CFO estimate tracks the ground truth over the full CFO range ($R^2 = 0.9986$, RMSE *= 0.0087*; Fig. 2a), providing a reliable input for frequency-offset compensation. For a representative channel with small CFO estimation error, the estimated CSI follows the ground-truth response across all active subcarriers, preserving deep fading notches in magnitude and the wrapped phase structure (Fig. 2b and c). Symbol reconstruction alone therefore supervises the unobservable CFO and CSI used by the receiver.

**Figure 2.**
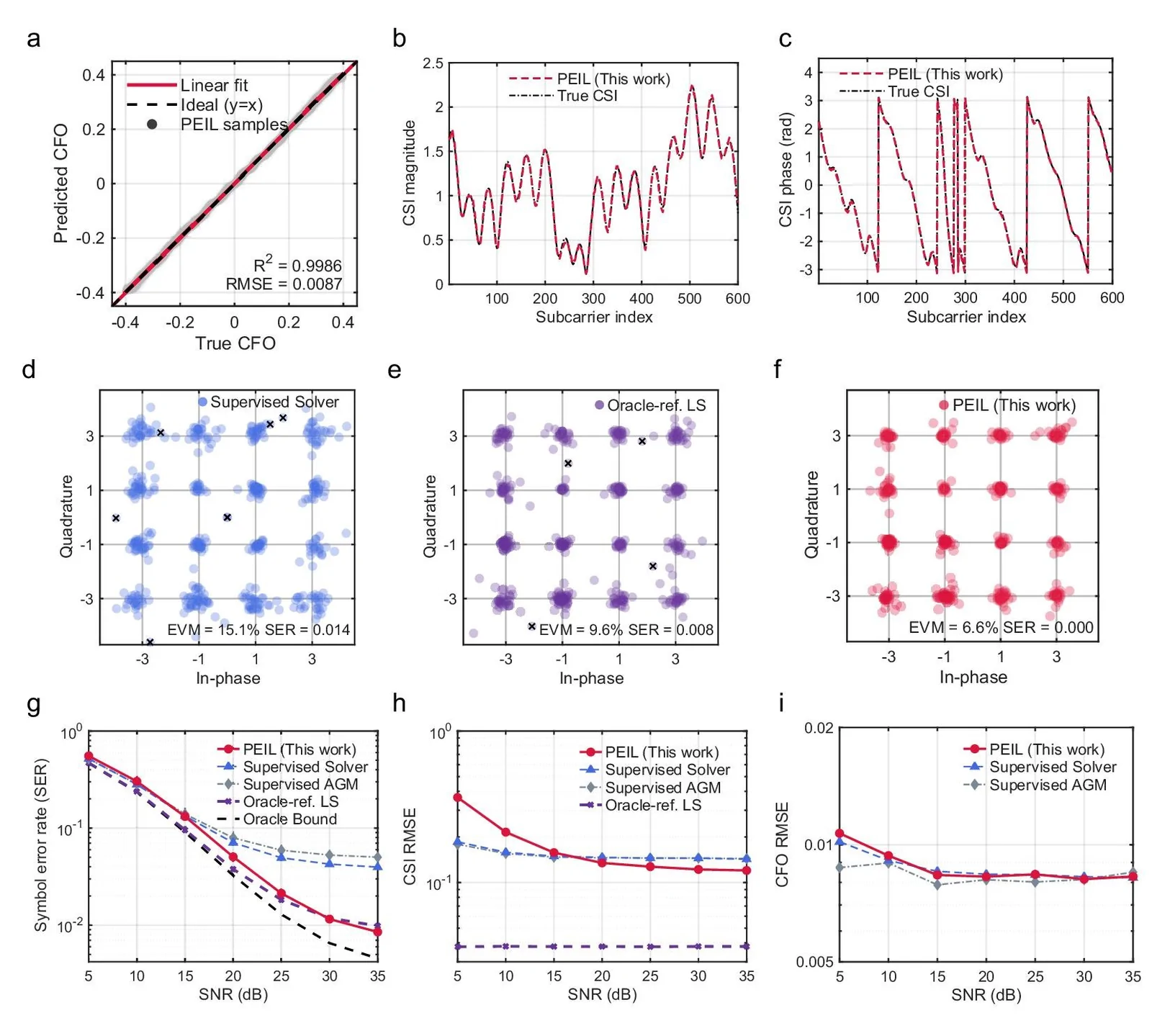
Wireless parameter estimation and symbol recovery under EVA fading with CFO. (a–c) Parameter estimates learned through symbol reconstruction. PEIL’s scalar CFO estimates closely follow the ground truth across the test set ($R^2 = 0.9986$, root mean square error (RMSE) *= 0.0087*; (a)). For a representative channel with small CFO estimation error, the estimated CSI follows the true CSI in magnitude, including deep fading notches (b), and in wrapped phase (c). (d–f) Symbol recovery from the same received snapshot at 30 dB SNR; black crosses mark symbol errors. Supervised Solver yields a dispersed constellation (error vector magnitude (EVM) = 15.1%, SER = 0.014; (d)). Oracle-ref. LS tightens the clustering but retains residual distortion (EVM = 9.6%, SER = 0.008; (e)). PEIL produces the tightest clustering (EVM = 6.6%, SER = 0 on this snapshot; (f)). (g–i) Performance across SNR. PEIL achieves lower SER than Oracle-ref. LS beyond 30 dB (g), despite a CSI RMSE above the oracle-reference interpolation floor (h) and nonzero CFO RMSE (i).

Figure 2d and f shows the recovery produced by these conditioning values for the same received snapshot at 30 dB SNR. Supervised Solver leaves a dispersed constellation, whereas Oracle-ref. LS produces tighter clusters but retains visible distortion. PEIL produces the most compact clusters and the clearest symbol separation. Because Supervised Solver shares PEIL’s estimator backbone and fixed receiver, their contrast shows that reconstruction supervision yields cleaner recovery than direct parameter supervision in this snapshot. The comparison with Oracle-ref. LS further shows that exact CFO and true pilot-channel values need not be the best inputs for recovery after fixed interpolation. Together, the two comparisons show that PEIL selects conditioning values by the symbols they recover rather than by their agreement with parameter labels.

Across the full test set, PEIL’s recovery advantage emerges as SNR increases (Fig. 2g). The supervised baselines retain a slight SER advantage below 15 dB but flatten above 20 dB, while PEIL continues to improve and falls below both. At high SNR, PEIL attains lower CSI RMSE than both supervised baselines, while CFO RMSE remains comparable across the learned methods (Fig. 2h and i). Beyond 30 dB, PEIL achieves lower SER than Oracle-ref. LS, although Oracle-ref. LS uses exact CFO and ground-truth pilot-channel values and attains the lowest CSI RMSE. These results capture PEIL’s task-optimal criterion. Under a fixed solver, conditioning selected through reconstruction can yield better recovery than nominally more accurate parameter inputs.

### Learning within a local non-identifiable set and adaptive gating

PEIL’s CFO and CSI errors vary together along a direction within a local reconstruction-level non-identifiable set. When the CFO error is small, PEIL’s CSI RMSE remains close to the oracle-reference interpolation floor, indicating high accuracy in both estimates (Fig. 3a). As $|\Delta \theta |$ increases, the CSI RMSE also rises. Figure 3b shows that PEIL’s CSI phase adjustment follows the local theoretical slope, whereas the supervised estimators exhibit steeper responses. The increasing CSI error therefore accompanies a phase adjustment that compensates for the residual CFO mismatch.

**Figure 3.**
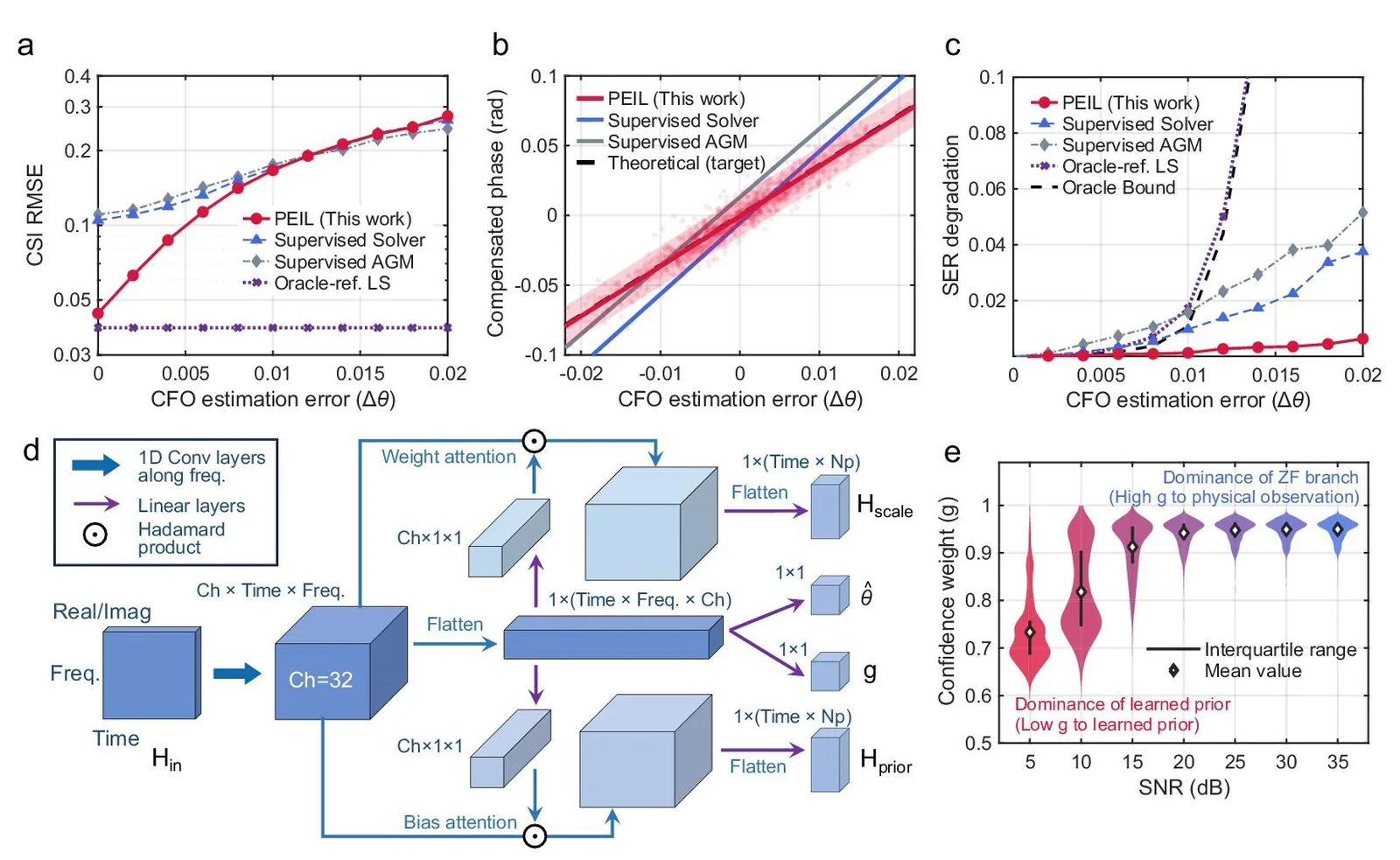
CFO–CSI behaviour within a local non-identifiable set and adaptive gating. For learned methods, $\Delta \theta$ is the observed CFO estimation error. Panels (a) and (c) use its magnitude, whereas panel (b) retains its sign. The reference curves impose CFO mismatch while holding CSI fixed, removing the corresponding CSI response. (a) CSI RMSE conditioned on $|\Delta \theta |$. Oracle-ref. LS (purple, flat) is unchanged because its CSI is held fixed. At small CFO error, both supervised baselines have higher CSI RMSE, whereas PEIL (red) lies near the oracle-reference interpolation floor; PEIL’s CSI RMSE then increases with CFO error. (b) Local phase direction. PEIL’s CSI phase adjustment (red) follows the local theoretical slope (black dashed), whereas the supervised methods exhibit steeper phase responses. (c) Reconstruction stability. PEIL’s SER degradation remains small across the observed CFO error range, whereas the supervised methods show greater degradation. With CSI held fixed, imposed CFO mismatch causes SER to degrade sharply in both reference curves. (d) Wireless estimator and gate output. From the pilot-grid input $\mathbf {H}_{\mathrm{in}}$, the estimator predicts $\!\!\hat{\,\,\,\theta }$, *\rm g*, $\mathbf {H}_{\mathrm{scale}}$, and $\mathbf {H}_{\mathrm{prior}}$. (e) Predicted gate across SNR. For the same trained model, which receives no explicit SNR input, *\rm g* is lower at 5–10 dB and approaches a plateau near 0.95 above 20 dB.

This local direction follows from the dominant noiseless residual CFO model $Y_{k,t}=H_{k,t}X_{k,t}e^{j\phi _t(\theta )}$, where *θ* is the true scalar CFO and $\phi _t(\theta )$ is its induced phase at time index *t*. Let $\!\!\hat{\,\,\,\theta }$ denote the estimated CFO and define the signed error as $\Delta \theta =\theta -\!\!\hat{\,\,\,\theta }$. The rotated channel is $H^{\prime }_{k,t}=H_{k,t}e^{j[\phi _t(\theta )-\phi _t(\!\!\hat{\,\,\,\theta })]}$. It satisfies $H^{\prime }_{k,t}e^{j\phi _t(\!\!\hat{\,\,\,\theta })}=H_{k,t}e^{j\phi _t(\theta )}$, so the fixed equaliser returns $\hat{X}_{k,t}=Y_{k,t}e^{-j\phi _t(\hat{\theta })}/H^{\prime }_{k,t}=X_{k,t}$. Define the CSI phase adjustment as $\Delta \psi _{H,t}=\arg (H^{\prime }_{k,t}/H_{k,t})$. To first order, $\Delta \psi _{H,t}\approx (\partial \phi _t/\partial \theta )\Delta \theta$, matching the local slope in Fig. 3b. This equivalence is exact in the local noiseless multiplicative model but approximate in the full receiver, where noise, interpolation, and residual inter-carrier interference remain.

Along this local direction, PEIL maintains stable symbol recovery across its actual CFO–CSI outputs (Fig. 3c). SER degradation is negligible for $|\Delta \theta |< 0.01$ and remains small as the error increases further, whereas the supervised methods degrade much more rapidly. The reference curves instead impose CFO mismatch while holding CSI fixed; without the corresponding CSI response, SER rises sharply beyond $|\Delta \theta |=0.01$. Figure 3 b identifies the compensating phase relation, and Fig. 3 c shows its effect on symbol recovery. Together, they show that PEIL learns coordinated CFO–CSI estimates rather than minimising the two errors independently. Because the fixed solver contains no learned correction, reconstruction loss selects the CFO–CSI pairs that best preserve the recovered symbols. This is task-optimal selection within the local non-identifiable set.

A second learned behaviour appears in the confidence gate [37], which mixes the scaled zero-forcing (ZF) and learned channel terms (Fig. 3d). The estimator receives no explicit SNR input, but the confidence gate (g) varies systematically across test samples from the same trained model. Its mean rises from approximately 0.73 at 5 dB to a plateau near 0.95 above 20 dB (Fig. 3e). At low SNR, the smaller gate assigns relatively more weight to the learned channel term; as pilot quality improves, the rising gate shifts the mixture towards the scaled ZF term. This adaptive mixture emerges through symbol reconstruction without explicit SNR supervision or a prescribed schedule.

### Robust generalisation and data efficiency

All models are trained exclusively on medium-complexity EVA channels at 120 km/h. Without retraining, we evaluate them on in-distribution EVA, unseen channel structures, and unseen velocities [38]. Training through the fixed receiver is expected to reduce reliance on statistical regularities specific to the training distribution. EPA tests whether the estimator avoids imposing training-specific structure on a simpler channel; ETU tests whether it can represent finer structure in a more complex one. On EPA, Supervised Solver introduces high-frequency fluctuations absent from the true channel, whereas PEIL follows the true channel’s smooth trend more closely. On ETU, Supervised Solver over-smooths the response and misses deep fading notches, whereas PEIL captures more of the true channel’s local variation (Fig. 4b and c). These differences are reflected in SER (Fig. 4a). On EPA and EVA, PEIL’s SER falls below both supervised baselines from moderate SNR onwards. The separation is more pronounced on ETU. Both supervised baselines remain at high SER and improve only modestly as SNR increases. Their weak SNR response indicates that their errors are dominated by structural mismatch rather than by measurement noise alone. PEIL, by contrast, continues to benefit from cleaner observations, reducing its SER from 0.68 at 5 dB to 0.24 at 35 dB. Together, the CSI profiles and SER trends show that PEIL generalises to both simpler and more complex channel structures without retraining.

**Figure 4.**
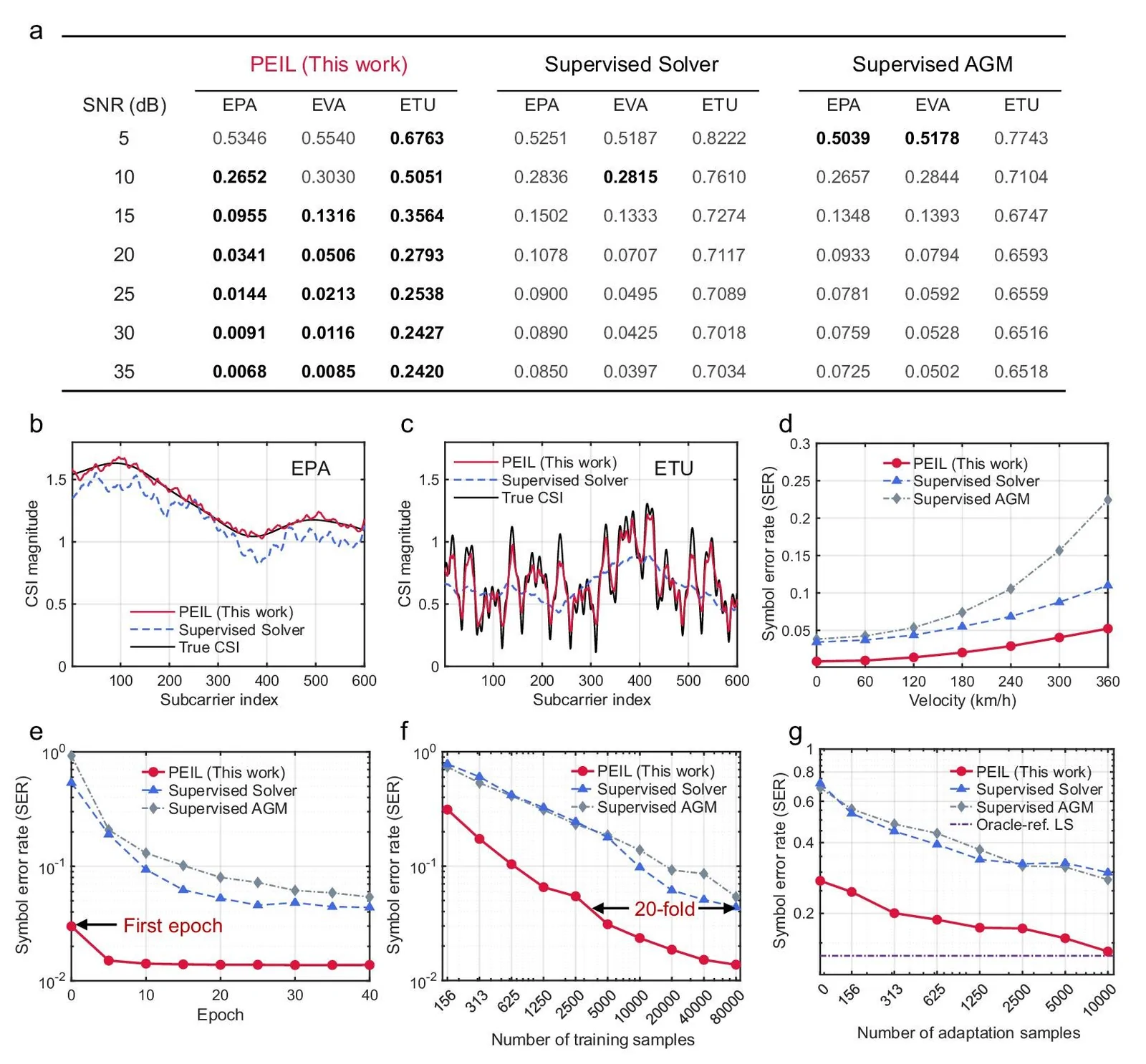
Generalisation and data efficiency across channel conditions. (a) Generalisation without retraining. Models trained on medium-complexity EVA dynamics at 120 km/h are tested on the in-distribution EVA setting and on unseen EPA and ETU channel structures. PEIL achieves lower SER than both supervised baselines from moderate SNR onwards on EPA and EVA and across all tested SNRs on ETU. (b, c) CSI profiles on unseen regimes. Supervised Solver introduces high-frequency artefacts in simple EPA channels (b) and over-smooths complex ETU channels, missing deep fading notches (c). PEIL follows the true spectral structure more closely in both cases. (d) Velocity-only extrapolation beyond the 120 km/h training condition. PEIL degrades gradually up to 360 km/h, whereas the supervised baselines degrade more rapidly. (e) Convergence trajectories. PEIL reaches a low validation SER within the first epoch, whereas both supervised baselines converge more slowly and remain at higher error floors. (f) Training-set efficiency. PEIL uses source signals rather than ground-truth CFO/CSI labels for supervision. With 4,000 training samples, it reaches the SER that the supervised baselines require 80,000 parameter-labelled samples to achieve, a 20-fold reduction in training samples. (g) Fine-tuning under extreme compound shift (high-mobility ETU). Without samples from the target condition, PEIL starts at SER *≈* 0.28, whereas both supervised methods start at SER *≥ 0.70*. They require 10,000 adaptation samples to approach PEIL’s performance without adaptation; with the same 10,000 samples, PEIL approaches Oracle-ref. LS.

PEIL’s advantage persists when velocity alone is increased beyond the 120 km/h training condition (Fig. 4d). At 360 km/h, PEIL’s SER remains near 0.05, compared with approximately 0.11 for Supervised Solver and 0.22 for Supervised AGM. These structural and kinematic results are consistent with the fixed receiver acting as a physical inductive bias. Parameter supervision optimises agreement with nominal values in the training distribution, whereas PEIL is trained by symbol recovery through that receiver.

Using symbol reconstruction to supervise the unobservable parameters removes the need for ground-truth CFO and CSI labels while improving data efficiency (Fig. 4e and f). PEIL reaches a low validation SER within the first epoch, whereas the parameter-supervised baselines converge more slowly and remain at higher error floors (Fig. 4e). With 4,000 training samples, PEIL reaches the SER that the baselines require 80,000 parameter-labelled samples to achieve (Fig. 4f). Compared with parameter-supervised training, this is a 20-fold reduction in the number of training samples.

PEIL’s out-of-distribution performance also provides a stronger starting point for adaptation to the target condition (Fig. 4g). Under the extreme compound shift of 360 km/h ETU, PEIL begins at SER *≈ 0.28* without fine-tuning on the target condition, whereas both parameter-supervised methods begin at SER *≥ 0.70*. To approach PEIL’s SER before adaptation, the supervised methods require 10,000 adaptation samples. With the same 10,000 samples from the target condition, PEIL approaches Oracle-ref. LS. Taken together, PEIL generalises without retraining, matches the parameter-supervised baselines with 20-fold fewer training samples, and begins adaptation to the target condition from a substantially lower error.

### Application to blind calibration in parallel MRI

The MRI experiment tests whether the same principle of discovering unobservable parameters through signal recovery extends to a different inverse problem. We use the multi-coil fastMRI knee dataset [39]. Here, the source image and coil sensitivities enter the forward model multiplicatively, with a scale–phase ambiguity distinct from the CFO–CSI coupling in wireless channels. PEIL estimates coil sensitivities without sensitivity-map labels or a separate calibration step and uses them to condition a fixed conjugate gradient (CG) solver. In this regularised instantiation, the solver also receives an image-domain reference $\mathbf {X}_{\mathrm{prior}}$ and a regularisation weight; $\mathbf {X}_{\mathrm{prior}}$ is an auxiliary input, whereas the CG solution is the reported reconstruction. The central test is whether image reconstruction through this fixed solver can supervise coil sensitivity estimation under a different physical coupling. TV-SENSE [14] provides a physical reconstruction reference with calibrated sensitivities, and Deep J-Sense [40] is the closest comparator for joint estimation. A supervised U-Net provides a reference for direct image prediction, and VarNet [41] provides a high-capacity learned reconstruction reference. All methods are evaluated under the same fastMRI protocol (Fig. 5a; full metrics in Table S1).

**Figure 5.**
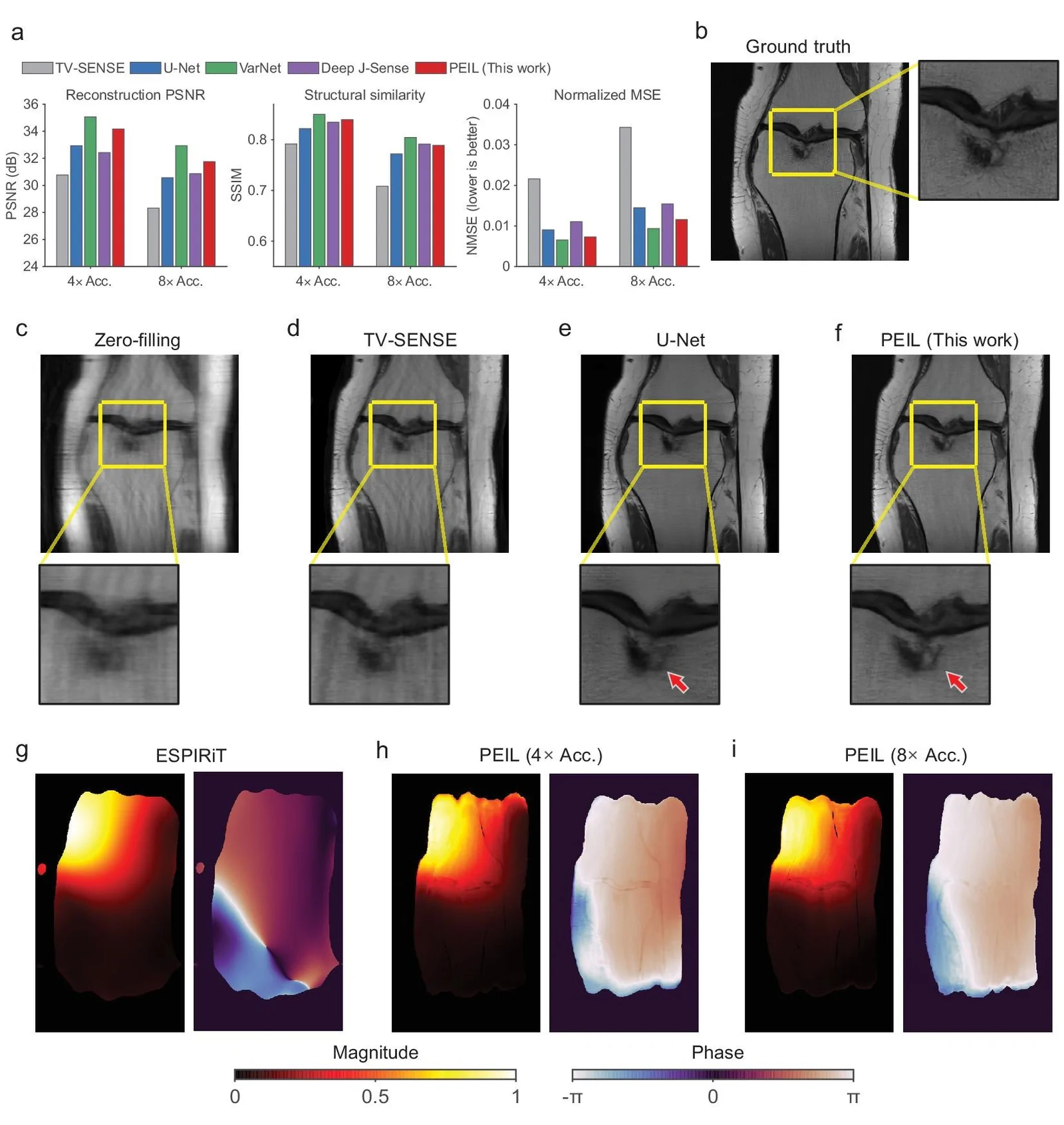
Blind calibration and image reconstruction in parallel MRI. (a) Reconstruction fidelity on the fastMRI knee dataset at *4×* and *8×* acceleration, measured by peak signal-to-noise ratio (PSNR), structural similarity index (SSIM), and normalised mean-squared error (NMSE). Without sensitivity-map labels or a separate calibration step, PEIL (red) achieves higher PSNR and lower NMSE than Deep J-Sense, the supervised U-Net, and TV-SENSE at both accelerations, with SSIM comparable to Deep J-Sense. VarNet achieves higher PSNR and lower NMSE. Full results are listed in Table S1. (b–f) Representative visual reconstruction at *8×* acceleration. Zero-filling (c) exhibits aliasing and blur; TV-SENSE (d) reduces aliasing but retains residual streaking and noise amplification; the supervised U-Net (e) shows texture smoothing. Compared with the displayed baselines, PEIL (f) better preserves sharp meniscal boundaries and fine trabecular textures (red arrows), more closely resembling the fully-sampled reference (b). (g–i) Blind coil sensitivity estimation. Magnitude and phase for one receiver coil are shown for ESPIRiT (calibration reference; (g)) and PEIL under *4×* (h) and *8×* (i) acceleration. Up to the scale–phase ambiguity of coil maps, the two PEIL estimates show consistent dominant magnitude and phase profiles, while their magnitude structure follows the calibration reference.

PEIL couples blind calibration with high-quality anatomical reconstruction through the fixed CG solver (Fig. 5a–f). Without sensitivity-map labels or a separate calibration step, it achieves higher PSNR and lower NMSE than Deep J-Sense, the supervised U-Net, and TV-SENSE at both acceleration factors. VarNet, a strong learned reconstruction baseline, uses 12 learned cascades and 29.94M learnable parameters to iteratively refine the image and achieves higher PSNR and lower NMSE. PEIL instead predicts the solver-conditioning quantities in a single forward pass with a 0.51M-parameter estimator, after which the fixed CG solver reconstructs the image. Its PSNR remains within 1.2 dB of VarNet’s at both acceleration factors. In the representative *8×* reconstruction, zero-filling exhibits aliasing and blur, TV-SENSE retains residual streaking and noise amplification, and the supervised U-Net shows texture smoothing. Compared with these displayed baselines, PEIL better preserves sharp meniscal boundaries and fine trabecular bone textures (Fig. 5b–f).

The estimated coil maps provide the direct test of blind calibration (Fig. 5g–i). For a given acquisition, coil sensitivity profiles should remain consistent across undersampling rates, up to the usual scale–phase ambiguity with the reconstructed image. Without sensitivity-map supervision, the PEIL maps at *4×* and *8×* acceleration retain consistent dominant magnitude and phase profiles, while their magnitude structure follows the ESPIRiT [42] calibration reference. The cross-acceleration consistency and magnitude correspondence with the calibration reference support the recovery of stable coil-dependent structure without sensitivity-map labels.

The fixed CG inversion contributes substantially beyond the network’s image-domain reference. We compare the final reconstruction with $\mathbf {X}_{\mathrm{prior}}$, which is produced by the estimator before physical inversion. Conditioned on the estimated coil sensitivities and using $\mathbf {X}_{\mathrm{prior}}$ as a regularisation reference, the CG solver reconstructs the image from acquired k-space. Relative to $\mathbf {X}_{\mathrm{prior}}$ alone, the final reconstruction improves PSNR by 5.5–6.1 dB and reduces NMSE by 67.7–75.2% at both acceleration factors (Table S2). Together, the stable coil maps and the gains from fixed inversion show that PEIL’s learned quantities effectively condition a fixed, data-fidelity-regularised solver. Under this distinct physical coupling, a fixed inverse solver again turns signal recovery into supervision for otherwise unobservable calibration quantities.

## DISCUSSION

PEIL demonstrates that signal recovery can supervise the estimation of unobservable physical quantities without parameter labels. In wireless channels, reconstruction training yields task-optimal conditioning values that improve symbol recovery. The learned CFO–CSI deviations also follow a compensatory direction within a local non-identifiable set. In MRI, PEIL learns stable coil sensitivity maps without sensitivity-map labels or a separate calibration step, and the fixed CG solver uses these maps in a data-fidelity-regularised reconstruction. Across both domains, signal recovery supervises the quantities required by the inverse solver.

Gradients from the reconstruction loss pass through the fixed solver to train the estimator. The estimator affects the final reconstruction through the quantities that condition that solver. Because no learned module corrects the output after inversion, the joint effect of these quantities is evaluated directly by the reconstruction loss. This does not require each predicted quantity to be independently accurate: the effect of an error in one may be partially offset by the others. Other physics-guided approaches give learned components more control over reconstruction. Physics-informed networks impose physics through soft penalties balanced against data fidelity [28,29]; many unrolled and bilevel formulations use learned solver updates or regularisers [20–25]; and self-supervised reconstruction methods learn reconstruction mappings or regularisers without fully sampled references [43]. This flexibility can correct modelling errors, but where conditioning quantities are estimated, learned reconstruction components can also compensate for their inaccuracies. PEIL instead keeps the inversion fixed, so any such compensation must act through the explicit conditioning quantities and their interaction within the solver.

A task-optimal setting need not coincide with physical truth. When the estimated parameters are themselves the quantities of interest, as when channel state is reused for sensing, deviations that improve reconstruction may be undesirable. Non-identifiability is most useful when the ambiguity is local and structured, allowing reconstruction loss to select among nearby representatives with little change to the recovered signal. Global or discrete ambiguities may require additional constraints or search mechanisms.

These results also have direct consequences for deployment. In wireless communications, PEIL reaches comparable error floors with 20-fold fewer training samples and generalizes across unseen channel profiles and velocities without retraining. This combination reduces the data and retraining burden as propagation conditions change. The observed robustness is consistent with the fixed receiver acting as a physical inductive bias, although transferable statistical regularities may also contribute. In MRI, estimating coil sensitivities without separate calibration can simplify reconstruction and reduce dependence on dedicated calibration data. More broadly, the PEIL structure can extend to inverse problems where a reliable fixed solver and references for the source signal are available, but direct labels for its conditioning quantities are not. Potential applications include optical channel equalisation, acoustic source localisation, and sensor array calibration.

The solver defines the main implementation boundary. The inverse need not have a closed-form solution; an iterative solver suffices, as the fixed CG solver used for MRI shows. If no reliable differentiable solver can be constructed from the available physics, PEIL would require an approximate solver or a different formulation. Differentiating through the fixed solver also incurs computational cost. In the MRI instantiation, each training step backpropagates through the unrolled CG iterations, repeatedly applying the multi-coil encoding operator and its adjoint and storing the unrolled states. Training is therefore slower and more memory-intensive than a purely feedforward pass; the measured wall-clock and memory costs are reported in Table S3.

PEIL also requires measurements that retain sample-specific information. At low SNR, the supervised baselines hold a slight advantage and the lower predicted gate assigns relatively more weight to the learned channel term. If the estimator input contains no meaningful measurement structure, recovery depends mainly on statistical regularities learned from the training data rather than on sample-specific physical conditioning. Blind separation of the source signal and unobservable parameters may also require domain-specific regularisation; in MRI, for example, the double-well potential constrains the scaling ambiguity between coil sensitivities and the image. Whether the gate learns the same SNR-dependent weighting in other domains remains open. A natural next step is to increase architectural flexibility around the fixed inversion. More expressive fixed-operator computational graphs could combine hard physical constraints with learned signal-domain priors while keeping the inversion step non-learnable.

## METHODS

### Problem formulation

The goal is to reconstruct a target signal $\mathbf {X} \in \mathbb {C}^{N}$ from indirect, noisy observations $\mathbf {Y} \in \mathbb {C}^{M}$ [44]. The forward process is governed by a domain-specific physical operator $\mathcal {F}$:


(1) \begin{eqnarray*}
\mathbf {Y} = \mathcal {P}_{\Omega } \left( \mathcal {F}(\mathbf {X}; \boldsymbol{\theta }, \mathbf {H}) \right) + \mathbf {n},
\end{eqnarray*}

where $\mathcal {P}_{\Omega }$ denotes the domain-specific observation pattern and $\mathbf {n}$ is additive noise. The required parameters $(\boldsymbol{\theta }, \mathbf {H})$ are unknown. In wireless systems, $\boldsymbol{\theta }$ reduces to the scalar CFO *θ*, and $\mathbf {H}$ is the instantaneous channel state. In parallel MRI, $\mathbf {H}$ corresponds to the coil sensitivity maps $\mathbf {S}$, with no corresponding system parameter. Recovery requires jointly estimating these parameters and inverting the physical operator without ground-truth parameter supervision. The formulation is related to blind deconvolution and calibration-free reconstruction [18,45], but uses an explicit physical operator rather than a purely data-driven prior.

We use non-identifiability in a local reconstruction-level sense. For a fixed observation, solver-conditioning settings are locally non-identifiable when they lie in a prescribed neighbourhood and their reconstructions differ by no more than a tolerance *δ*. Settings in this set need not be physically identical; they recover nearly the same signal through the chosen inverse operator. The definition is specific to that operator and reconstruction task and does not imply global physical equivalence. The formal definition is provided in Method S1.

### Estimator and fixed inverse solvers

PEIL follows an estimate-then-solve structure. The estimator input is derived from the observations and is chosen to retain sample-specific features informative about the required solver-conditioning quantities. A fixed, non-learnable solver then uses those quantities to reconstruct the target signal. In wireless systems, the estimator predicts CFO and CSI-related quantities for a fixed receiver. In MRI, it predicts coil sensitivities together with the image-domain reference and regularisation weight required by a fixed CG solver.

The MRI implementation uses a U-Net backbone and a 15-iteration fixed CG solver [46,47]. Complete estimator and solver formulations are provided in Method S2–S4, with layer specifications in Tables S4–S6.

### Training

Although the required parameters $(\boldsymbol{\theta }, \mathbf {H})$ are unknown, a reference for the target signal is available during training. The model is trained end-to-end by minimising


(2) \begin{eqnarray*}
\mathcal {L} = \mathcal {L}_{\mathrm{rec}} + \gamma \mathcal {L}_{\mathrm{phys}}.
\end{eqnarray*}

For wireless systems, $\mathbf {X}_{\mathrm{gt}}$ contains the transmitted complex symbols and $\mathcal {L}_{\mathrm{rec}}=\Vert \!\!\hat{\mathbf {\,\,\,X}}-\mathbf {X}_{\mathrm{gt}}\Vert _1$, where $\Vert \mathbf {z}\Vert _1=\sum _k|z_k|$ uses the complex modulus. Here *γ =0*, so the estimator is trained solely by the symbol-reconstruction loss propagated through the fixed receiver. For MRI, $\mathbf {X}_{\mathrm{gt}}$ is the official fastMRI root-sum-of-squares (RSS) magnitude target, and $\mathcal {L}_{\mathrm{rec}}=\Vert |\!\!\hat{\mathbf {\,\,\,X}}|-\mathbf {X}_{\mathrm{gt}}\Vert _1$. We use *γ =0.1* for MRI; the multi-coil consistency term [40] and double-well term are given in Method S5. Neither term requires ground-truth coil sensitivity labels, and no separate loss is applied to $\mathbf {X}_{\mathrm{prior}}$.

### Experimental details

We simulate a 3GPP LTE-FDD OFDM downlink under EPA, EVA, and ETU channel models [33], with a 2.6 GHz carrier, 10 MHz bandwidth, FFT length 1024, cyclic prefix (CP) length 72, and random CFO. We use the multi-coil fastMRI knee dataset [39] with Cartesian undersampling at *4×* and *8×* acceleration. PEIL is trained with the Adam optimiser [48] in both domains. Additional experimental details are provided in Method S6.

## Supplementary Material

nwag508_Supplemental_File

## Data Availability

The wireless communication data generated in this study are based on standard 3GPP LTE-FDD simulations using EPA, EVA, and ETU channel models as detailed in the Methods section and Method S6. Source data supporting the findings of this study are available from the corresponding author upon reasonable request. The MRI data used in this study are derived from the publicly available fastMRI knee dataset, which can be accessed at https://fastmri.med.nyu.edu/ upon registration.

## References

[1] You X, Wang CX, Huang J et al. Towards 6G wireless communication networks: vision, enabling technologies, and new paradigm shifts. Sci China Inf Sci 2021; 64: 110301. 10.1007/s11432-020-2955-6

[2] Zhu B, Liu JZ, Cauley SF et al. Image reconstruction by domain-transform manifold learning. Nature 2018; 555: 487–92. 10.1038/nature25988 29565357

[3] Hammernik K, Klatzer T, Kobler E et al. Learning a variational network for reconstruction of accelerated MRI data. Magn Reson Med 2018; 79: 3055–71. 10.1002/mrm.26977 PMC5902683 29115689

[4] Arridge S, Maass P, Öktem O et al. Solving inverse problems using data-driven models. Acta Numer 2019; 28: 1–174. 10.1017/S0962492919000059

[5] McCann MT, Jin KH, Unser M. Convolutional neural networks for inverse problems in imaging: a review. IEEE Signal Process Mag 2017; 34: 85–95. 10.1109/MSP.2017.2739299 28641250

[6] Lucas A, Iliadis M, Molina R et al. Using deep neural networks for inverse problems in imaging: beyond analytical methods. IEEE Signal Process Mag 2018; 35: 20–36. 10.1109/MSP.2017.2760358

[7] LeCun Y, Bengio Y, Hinton G. Deep learning. Nature 2015; 521: 436–44. 10.1038/nature14539 26017442

[8] Liu J, Zhao H, Ma D et al. Theoretical analysis of deep neural networks in physical layer communication. IEEE Trans Commun 2022; 70: 6589–603. 10.1109/TCOMM.2022.3201931

[9] O’Shea T, Hoydis J. An introduction to deep learning for the physical layer. IEEE Trans Cogn Commun Netw 2017; 3: 563–75. 10.1109/TCCN.2017.2758370

[10] Tobin J, Fong R, Ray A et al. Domain randomization for transferring deep neural networks from simulation to the real world. In: IEEE/RSJ International Conference on Intelligent Robots and Systems (IROS), Vancouver, BC, Canada, 2017; 23–30.

[11] Feng X, Xiong J, Liu X et al. Physics-guided language model via low-rank adaptation for path loss prediction. IEEE Trans Cogn Commun Netw 2026; 12: 3179–90. 10.1109/TCCN.2025.3620359

[12] Kong L, Liu X, Zhang X et al. Representation-based continual learning for channel estimation in dynamic wireless environments. IEEE Trans Wireless Commun 2025; 24: 6382–96. 10.1109/TWC.2025.3552836

[13] Tse D, Viswanath P. Fundamentals of Wireless Communication. Cambridge: Cambridge University Press, 2005. 10.1017/CBO9780511807213

[14] Pruessmann KP, Weiger M, Scheidegger MB et al. SENSE: sensitivity encoding for fast MRI. Magn Reson Med 1999; 42: 952–62. 10.1002/(SICI)1522-2594(199911)42:5<952::AID-MRM16>3.0.CO;2-S 10542355

[15] de Avila Belbute-Peres FA, Smith KA, Allen KR et al. End-to-end differentiable physics for learning and control. In: Advances in Neural Information Processing Systems 2018; 31: 7178–89.

[16] Degrave J, Hermans M, Dambre J et al. A differentiable physics engine for deep learning in robotics. Front Neurorobot 2019; 13: 6. 10.3389/fnbot.2019.00006 PMC6416213 30899218

[17] Holl P, Thuerey N, Koltun V. Learning to control PDEs with differentiable physics. International Conference on Learning Representations, Virtual Conference, 26 April–1 May 2020.

[18] Choudhary S, Mitra U. Identifiability scaling laws in bilinear inverse problems [preprint]. arXiv: 1402.2637. arXiv:1402.2637

[19] Li Y, Lee K, Bresler Y. Identifiability in blind deconvolution with subspace or sparsity constraints. IEEE Trans Inf Theory 2016; 62: 4266–75. 10.1109/TIT.2016.2569578

[20] Gilton D, Ongie G, Willett R. Deep equilibrium architectures for inverse problems in imaging. IEEE Trans Comput Imaging 2021; 7: 1123–33. 10.1109/TCI.2021.3118944

[21] Grazzi R, Franceschi L, Pontil M et al. On the iteration complexity of hypergradient computation. In: Proceedings of the 37th International Conference on Machine Learning 2020; 119: 3748–58.

[22] Yu H, Fessler JA, Jiang Y. Bilevel optimized implicit neural representation for scan-specific accelerated MRI reconstruction. IEEE Trans Med Imaging 2026; 45: 3792–808. 10.1109/TMI.2026.3686724 PMC13160684 42019070

[23] Monga V, Li Y, Eldar YC. Algorithm unrolling: Interpretable, efficient deep learning for signal and image processing. IEEE Signal Process Mag 2021; 38: 18–44. 10.1109/MSP.2020.3016905

[24] Gregor K, LeCun Y. Learning fast approximations of sparse coding. In: Proceedings of the 27th International Conference on Machine Learning 2010; 399–406.

[25] Zhang J, Ghanem B. ISTA-Net: Interpretable optimization-inspired deep network for image compressive sensing. In: Proceedings of the IEEE/CVF Conference on Computer Vision and Pattern Recognition 2018; 1828–37.

[26] Jacome R, Gualdrón-Hurtado R, Suarez-Rodriguez L et al. UTOPY: Unrolling algorithm learning via fidelity homotopy for inverse problems. In: IEEE 10th International Workshop on Computational Advances in Multi-Sensor Adaptive Processing (CAMSAP), Punta Cana, Dominican Republic, 2025; 176–80.

[27] Shlezinger N, Whang J, Eldar YC et al. Model-based deep learning. Proc IEEE 2023; 111: 465–99. 10.1109/JPROC.2023.3247480

[28] Raissi M, Perdikaris P, Karniadakis GE. Physics-informed neural networks: a deep learning framework for solving forward and inverse problems involving nonlinear partial differential equations. J Comput Phys 2019; 378: 686–707. 10.1016/j.jcp.2018.10.045

[29] Karniadakis GE, Kevrekidis IG, Lu L et al. Physics-informed machine learning. Nat Rev Phys 2021; 3: 422–40. 10.1038/s42254-021-00314-5

[30] Hemachandra A, Lau GKR, Ng SK et al. PIED: physics-informed experimental design for inverse problems. In: International Conference on Learning Representations, Singapore, 24–28 April 2025.

[31] Jatavallabhula KM, Macklin M, Golemo F et al. gradSim: differentiable simulation for system identification and visuomotor control. In: International Conference on Learning Representations, Virtual Conference, 3–7 May 2021.

[32] Yang Y, Gao F, Ma X et al. Deep learning-based channel estimation for doubly selective fading channels. IEEE Access 2019; 7: 36579–89. 10.1109/ACCESS.2019.2901066

[33] 3GPP . LTE; evolved universal terrestrial radio access (E-UTRA); user equipment (UE) radio transmission and reception. 3GPP TS 36.101, version 18.6.0, Release 18, 2024.

[34] Coleri S, Ergen M, Puri A et al. Channel estimation techniques based on pilot arrangement in OFDM systems. IEEE Trans Broadcast 2002; 48: 223–9. 10.1109/TBC.2002.804034

[35] Chen Z, Liu Z, Geng X et al. Attention-guided multi-task network for joint CFO and channel estimation in OFDM systems. IEEE Trans Wireless Commun 2024; 23: 321–33. 10.1109/TWC.2023.3277561

[36] Kay SM . Fundamentals of Statistical Signal Processing: Estimation Theory. Englewood Cliffs, NJ: Prentice Hall, 1993.

[37] Kendall A, Gal Y. What uncertainties do we need in Bayesian deep learning for computer vision? In: Advances in Neural Information Processing Systems 2017; 30: 5574–84.

[38] Liu J, Shen Z, He Y et al. Towards out-of-distribution generalization: a survey [preprint]. arXiv: 2108.13624. arXiv:2108.13624

[39] Knoll F, Zbontar J, Sriram A et al. fastMRI: a publicly available raw k-space and DICOM dataset of knee images for accelerated MR image reconstruction using machine learning. Radiol Artif Intell 2020; 2: e190007. 10.1148/ryai.2020190007 PMC6996599 32076662

[40] Arvinte M, Vishwanath S, Tewfik AH et al. Deep J-Sense: accelerated MRI reconstruction via unrolled alternating optimization. In: Medical Image Computing and Computer-Assisted Intervention 2021; 12906: 350–60. 10.1007/978-3-030-87231-1_34 PMC8767765 35059693

[41] Sriram A, Zbontar J, Murrell T et al. End-to-end variational networks for accelerated MRI reconstruction. In: Medical Image Computing and Computer-Assisted Intervention 2020; 12262: 64–73.

[42] Uecker M, Lai P, Murphy MJ et al. ESPIRiT—an eigenvalue approach to autocalibrating parallel MRI: where SENSE meets GRAPPA. Magn Reson Med 2014; 71: 990–1001. 10.1002/mrm.24751 PMC4142121 23649942

[43] Yaman B, Hosseini SAH, Moeller S et al. Self-supervised learning of physics-guided reconstruction neural networks without fully sampled reference data. Magn Reson Med 2020; 84: 3172–91. 10.1002/mrm.28378 PMC7811359 32614100

[44] Donoho DL . Compressed sensing. IEEE Trans Inf Theory 2006; 52: 1289–306. 10.1109/TIT.2006.871582

[45] Gull SF, Daniell GJ. Image reconstruction from incomplete and noisy data. Nature 1978; 272: 686–90. 10.1038/272686a0

[46] Ronneberger O, Fischer P, Brox T. U-Net: convolutional networks for biomedical image segmentation. In: Medical Image Computing and Computer-Assisted Intervention 2015; 9351: 234–41.

[47] Hestenes MR, Stiefel E. Methods of conjugate gradients for solving linear systems. J Res Natl Bur Stand 1952; 49: 409–36. 10.6028/jres.049.044

[48] Kingma DP, Ba J. Adam: a method for stochastic optimization. In: International Conference on Learning Representations, San Diego, CA, USA, 7–9 May 2015.

