# Supplementary material for: Discovery of unobservable parameters via physical embedding
Source: Natl Sci Rev. 2026 Aug 19;13(18):nwag508. doi: 10.1093/nsr/nwag508 (PMC13625021; doi:10.1093/nsr/nwag508)
Supplement: Supplementary file 1 [file nwag508_supplemental_file.pdf]

Supplementary Data

Discovery of unobservable parameters via physical embedding

Le Cheng, Xiaoran Liu, Lingjin Kong, Haitao Zhao, Jun Xiong, Fanglin Gu, Xiaoying Zhang, Baoquan Ren, Jibo Wei, Hao Yin

This file provides the detailed methods, supplementary quantitative comparisons, prior-to-final reconstruction measurements, computational costs, and estimator specifications referenced in the main manuscript. References for named baseline methods are provided in the main text.

Supplementary Methods

S1. Problem formulation and local non-identifiability

The goal is to reconstruct a target signal $\mathbf{X} \in \mathbb{C}^N$ from indirect, noisy observations $\mathbf{Y} \in \mathbb{C}^M$. These observations are only partially informative. In MRI, k-space is undersampled; in OFDM, channel calibration relies on sparse pilots while the received data symbols remain noisy.

We use non-identifiability in a local reconstruction-level sense. Let $\boldsymbol{\eta}$ denote the quantities that condition the fixed solver. These may include physical calibration quantities, such as CFO/CSI or coil sensitivity maps, and auxiliary inputs required by a regularised solver, such as an image-domain reference or regularisation weight. Physical interpretation applies only to the calibration quantities. Let $\mathcal{R}(\mathbf{Y}; \boldsymbol{\eta})$ denote the reconstruction produced by the fixed solver, and let $\mathcal{U}(\boldsymbol{\eta}_0)$ denote a prescribed neighbourhood of a reference setting $\boldsymbol{\eta}_0$. For a fixed observation $\mathbf{Y}$ and tolerance $\delta > 0$, the local reconstruction-level non-identifiable set is

$$\mathcal{N}_\delta(\boldsymbol{\eta}_0; \mathbf{Y}) = \big\{\boldsymbol{\eta} \in \mathcal{U}(\boldsymbol{\eta}_0) : d(\mathcal{R}(\mathbf{Y}; \boldsymbol{\eta}), \mathcal{R}(\mathbf{Y}; \boldsymbol{\eta}_0)) \leq \delta\big\}, \tag{S1}$$

where $d(\cdot, \cdot)$ measures reconstruction discrepancy and δ sets the resolution at which two reconstructions are treated as nearly the same. Settings in this set need not be physically identical; they recover nearly the same signal through the chosen inverse operator. The definition is specific to that operator and reconstruction task and does not imply global physical equivalence.

S2. Neural estimators

In wireless systems, zero-forcing gives a pilot-grid estimator input $\mathbf{H}_{\text{in}}$, with entries $H_{\text{in},k,t} = Y_{k,t}/X^{\text{pilot}}_{k,t}$. Under the dominant residual-CFO model, this input preserves local signatures of CSI and CFO-induced phase. The estimator applies a frequency-axis convolutional stem followed by fully connected heads, and predicts a channel prior $\mathbf{H}_{\text{prior}}$, a multiplicative scaling factor $\mathbf{H}_{\text{scale}}$, the scalar CFO $\hat{\theta}$, and a direct confidence-gate logit a_{g}, with $\mathsf{g} = \sigma(a_{\mathsf{g}}) \in (0, 1)$. In MRI, the estimator input comprises zero-filled coil images obtained by inverse fast Fourier transform (FFT), which preserve coil-dependent modulation and sample-specific anatomy despite aliasing. The estimator uses a U-Net backbone and predicts the complex-valued coil sensitivity maps $\hat{\mathbf{S}}$, a de-aliased image-domain reference $\mathbf{X}_{\text{prior}}$, and a regularisation weight ρ_{CG}. Layer-by-layer estimator specifications and parameter counts are provided in Tables S4–S6.

S3. Fixed wireless receiver

The receiver first compensates for the estimated CFO using the residual-phase model, $\tilde{Y}_{k,t} = Y_{k,t} e^{-j\phi_t(\hat{\theta})}$, where $\phi_t(\hat{\theta})$ is the CFO-induced phase at time index t. At pilot locations, it then forms the CFO-corrected ZF branch $\mathbf{H}_{\text{ZF}}(\hat{\theta}) = \tilde{\mathbf{Y}}_{\text{pilot}} \oslash \mathbf{X}^{\text{pilot}}$. The dense CSI is recovered by fusing this branch with the learned prior through a fixed Gaussian interpolation kernel $\mathcal{K}$:

$$\hat{\mathbf{H}} = \mathcal{K}\left(\mathsf{g}\mathbf{H}_{\text{ZF}}(\hat{\theta}) \odot \mathbf{H}_{\text{scale}} + (1 - \mathsf{g})\mathbf{H}_{\text{prior}}\right), \tag{S2}$$

where the kernel width is fixed by the receiver configuration. The target signal is recovered by coherent equalisation: $\hat{\mathbf{X}} = \tilde{\mathbf{Y}} \oslash \hat{\mathbf{H}}$.

Let $\mathbf{Z} = \mathsf{g}(\mathbf{H}_{\mathrm{ZF}}(\hat{\theta}) \odot \mathbf{H}_{\mathrm{scale}}) + (1-\mathsf{g})\mathbf{H}_{\mathrm{prior}}$ denote the pre-interpolation fused channel. With complex quantities represented by real and imaginary channels, the reconstruction loss gives the local gradient

$$\frac{\partial \mathcal{L}_{\mathrm{rec}}}{\partial a_{\mathsf{g}}} = \mathsf{g}(1-\mathsf{g})\,\mathrm{Re}\left\langle \frac{\partial \mathcal{L}_{\mathrm{rec}}}{\partial \mathbf{Z}}, \mathbf{H}_{\mathrm{ZF}}(\hat{\theta}) \odot \mathbf{H}_{\mathrm{scale}} - \mathbf{H}_{\mathrm{prior}} \right\rangle, \tag{S3}$$

where $\mathcal{L}_{\mathrm{rec}}$ denotes the reconstruction term. For fixed branch outputs, this gradient supplies a reconstruction-dependent update to g. Empirically, the predicted gate is lower at low SNR and increases as SNR improves.

S4. Fixed MRI solver

The reconstruction is defined by the Tikhonov-regularised problem

$$\mathbf{X}^{\star} = \arg\min_{\mathbf{X}} \big(\|\mathbf{Y} - \mathcal{P}_{\Omega}(\mathbf{F}(\hat{\mathbf{S}} \odot \mathbf{X}))\|_2^2 + \rho_{\mathrm{CG}} \|\mathbf{X} - \mathbf{X}_{\mathrm{prior}}\|_2^2 \big), \tag{S4}$$

where $\mathbf{F}$ is the 2D FFT and the predicted weight ρ_{CG} is constrained to be positive. The neural estimator parameterises $\hat{\mathbf{S}}$, $\mathbf{X}_{\mathrm{prior}}$, and ρ_{CG}, but the solver itself has no learnable parameters. The image-domain reference $\mathbf{X}_{\mathrm{prior}}$, the weight ρ_{CG}, and the estimated coil sensitivities together condition the reconstruction, while the data-fidelity term remains tied to the acquired k-space. In practice, the fixed solver applies 15 conjugate-gradient iterations to the corresponding normal equations, initialised with $\mathbf{X}_{\mathrm{prior}}$. The resulting finite-iteration solution is the reported reconstruction $\hat{\mathbf{X}}$ and remains differentiable during training.

S5. Training objectives

Although the required parameters $(\boldsymbol{\theta}, \mathbf{H})$ are unknown, a reference for the target signal is available during training. The model is trained end-to-end by minimising

$$\mathcal{L} = \mathcal{L}_{\mathrm{rec}} + \gamma \mathcal{L}_{\mathrm{phys}}. \tag{S5}$$

For wireless systems, $\mathbf{X}_{\mathrm{gt}}$ contains the transmitted complex symbols and $\mathcal{L}_{\mathrm{rec}} = \|\hat{\mathbf{X}} - \mathbf{X}_{\mathrm{gt}}\|_1$, where $\|\mathbf{z}\|_1 = \sum_k |z_k|$ uses the complex modulus. Here $\gamma = 0$, so the estimator is trained solely by the symbol-reconstruction loss propagated through the fixed receiver. For MRI, $\mathbf{X}_{\mathrm{gt}}$ is the official fastMRI root-sum-of-squares (RSS) magnitude target, and $\mathcal{L}_{\mathrm{rec}} = \||\hat{\mathbf{X}}| - \mathbf{X}_{\mathrm{gt}}\|_1$. We use $\gamma = 0.1$ and

$$\mathcal{L}_{\mathrm{phys}} = \|\mathbf{Y} - \mathcal{P}_{\Omega}(\mathbf{F}(\hat{\mathbf{S}} \odot \mathbf{X}_{\mathrm{gt}}))\|_2^2 + \beta\, \mathrm{mean}\Big[(\hat{\mathbf{S}}_{\mathrm{rss}}(\hat{\mathbf{S}}_{\mathrm{rss}} - 1))^2 \Big], \tag{S6}$$

with $\beta = 5$. Here, $\hat{\mathbf{S}}_{\mathrm{rss}}$ denotes the root-sum-of-squares magnitude of the estimated coil sensitivities. In the multi-coil consistency term, the RSS target is treated as a real, non-negative image. The reconstruction loss penalises errors in the recovered image, while the multi-coil consistency term enforces agreement between the estimated coil maps and the acquired observations. Neither term requires ground-truth coil sensitivity labels, and no separate loss is applied to $\mathbf{X}_{\mathrm{prior}}$. The double-well potential is an MRI-specific regulariser that encourages the root-sum-of-squares sensitivity magnitude to approach either 0 (background) or 1 (object support), constraining the blind calibration problem and helping avoid degenerate coil-image decompositions.

In this work, task-optimality means selection for reconstruction through the fixed solver rather than agreement with nominal parameter labels. In MRI, this criterion is combined with the multi-coil consistency and double-well terms above.

S6. Experimental protocols

Wireless experiments. We simulate a 3GPP LTE-FDD OFDM downlink under EPA, EVA, and ETU channel models, with a 2.6 GHz carrier, 10 MHz bandwidth, FFT length 1024, cyclic prefix (CP) length 72, and random CFO. The receiver uses 600 active subcarriers and 100 uniformly spaced pilots. Dense CSI is obtained with a fixed frequency-axis Gaussian kernel of size $(13, 1)$ and $\sigma = 3.0$ (Table S5). CFO values are reported as fractions of the subcarrier spacing. Supervised Solver is trained using mean squared error on the ground-truth CFO and CSI labels.

MRI experiments. We use the multi-coil fastMRI knee dataset with Cartesian undersampling at 4× and 8× acceleration. All reported MRI metrics use the official fastMRI knee validation split. Separate models are trained for the two acceleration factors. For the cross-acceleration coil-map comparison, the same fully sampled acquisition is undersampled at both rates and processed by the corresponding models. Reconstructions are evaluated in magnitude against the official RSS target using PSNR, SSIM, and NMSE. PEIL is trained with the Adam optimiser in both domains; estimator architectures, parameter counts, and matched computational measurements are provided in Tables S3–S6.

Supplementary Tables

Table S1. Expanded MRI quantitative comparison on the fastMRI knee dataset.

Method	4× acceleration			8× acceleration		
	PSNR (dB)	SSIM	NMSE	PSNR (dB)	SSIM	NMSE
TV-SENSE	30.77	0.7918	0.021618	28.32	0.7083	0.034292
Supervised U-Net	32.93	0.8219	0.009039	30.57	0.7719	0.014453
VarNet	35.06	0.8502	0.006528	32.93	0.8043	0.009362
Deep J-Sense	32.42	0.8344	0.011071	30.86	0.7914	0.015410
PEIL	34.17	0.8395	0.007280	31.75	0.7887	0.011589

All MRI methods are evaluated on the official fastMRI knee validation split under the same Cartesian acceleration settings and 320×320 centre crop. Peak signal-to-noise ratio (PSNR), structural similarity index (SSIM), and normalised mean squared error (NMSE) are recomputed under the matched evaluation protocol. VarNet is evaluated in the multi-coil setting using the official fastMRI implementation with 12 cascades and 18 channels. Deep J-Sense denotes the joint sensitivity-estimation and reconstruction baseline. For learned methods, the reported results use checkpoints from completed training runs. TV-SENSE denotes TV-regularised sensitivity encoding.

Table S2. Final PEIL reconstruction compared with the image-domain reference $\mathbf{X}_{\text{prior}}$.

Acceleration	Output	PSNR (dB)	SSIM	NMSE	PSNR gain	NMSE reduction
4×	$\mathbf{X}_{\text{prior}}$	28.65	0.7911	0.022543	–	–
4×	Final PEIL	34.17	0.8395	0.007280	+5.52 dB	67.7%
8×	$\mathbf{X}_{\text{prior}}$	25.69	0.7383	0.046786	–	–
8×	Final PEIL	31.75	0.7887	0.011589	+6.06 dB	75.2%

The final PEIL image is obtained by the fixed CG solver using the estimated coil sensitivities, acquired k-space data, and $\mathbf{X}_{\text{prior}}$ as the Tikhonov reference.

Table S3. Computational costs for MRI and wireless experiments.

MRI benchmark.

Method	Parameters (M)	Inference (ms/slice)	Peak inference memory (MB)	Training step (ms)	Peak training memory (MB)
PEIL	0.51	18.70 ± 0.27	770.6	70.07 ± 0.12	8158.0
Supervised U-Net	8.57	4.78 ± 0.06	386.2	13.90 ± 0.02	729.5
VarNet	29.94	47.90 ± 0.40	733.2	153.03 ± 0.50	8309.1
Deep J-Sense	0.45	137.04 ± 0.66	513.7	245.62 ± 6.03	9063.9
Zero-filling	0.00	2.28	227.1	–	–
TV-SENSE	0.00	714.80	269.6	–	–

Wireless benchmark.

Method	Active parameters	Inference (ms/batch)	Peak inference memory (MB)	Training step (ms/batch)	Peak training memory (MB)
PEIL	23,972,098	0.942	144.2	4.714	534.7
Supervised Solver	27,207,521	0.816	193.1	4.582	612.1
Supervised AGM	3,985,297	0.928	98.5	3.061	162.1
Oracle-ref. LS	0	0.257	81.6	–	–

These are practical wall-clock and memory measurements under matched hardware and precision, rather than FLOP-normalised complexity counts. MRI measurements were run with batch size 1 on an NVIDIA GeForce RTX 5090 D using PyTorch 2.12.0/CUDA 13.2 in FP32. Inference time is measured per validation slice after warmup. A training step includes forward propagation, reconstruction loss, backpropagation, and one optimiser update. MRI times are reported as mean $\pm$ standard deviation across the measured inference samples or training steps. Wireless measurements used the same hardware and precision with same-shape synthetic inputs, batch size 64, 5 warmup iterations, and 20 timed iterations. PEIL and Supervised Solver share the estimator backbone, while their task-specific output heads differ. The active wireless parameter count includes only modules used in the reported forward path. Dashes denote non-trainable classical baselines for which a training-step measurement is not applicable.

Table S4. PEIL architecture overview and parameter counts.

Model	Input	Learned estimator	Fixed inverse step	Active trainable estimator parameters
PEIL in wireless	Pilot-grid zero-forcing (ZF) input $\mathbf{H}_{\text{in}}$ with shape $[B, 2, 100, 4]$	Frequency-axis Conv2d stem $2 \to 32 \to 32$ with kernel $(7, 1)$ and LeakyReLU; CFO MLP $11264 \to 512 \to 256 \to 128$; heads for CFO, $\mathtt{g} = \sigma(a_{\mathtt{g}})$, $\mathbf{H}_{\text{scale}}$, and $\mathbf{H}_{\text{prior}}$	CFO compensation, fixed Gaussian interpolation, and equalisation	23,972,098 active estimator parameters
PEIL in MRI	Coil-wise zero-filled complex images $[B, N_c, 2, H, W]$, reshaped to $[B, 2N_c, H, W]$ with $N_c = 15$	Two-level U-Net-style encoder-decoder with channels 48/96/192, 3×3 convolutions, SiLU/LayerNorm blocks, and squeeze-and-excitation (SE) blocks; heads for sensitivity phase/amplitude, $\mathbf{X}_{\text{prior}}$ phase/amplitude, and ρ_{CG}	15-iteration fixed CG solver	507,009 estimator parameters

The fixed Gaussian interpolator and the fixed CG solver have no trainable parameters. Detailed parameter breakdowns are given in Tables S5 and S6.

Table S5. PEIL architecture breakdown for wireless.

Component	Layers or operation	Output shape	Activation or constraint	Parameters
Estimator input $\mathbf{H}_{\text{in}}$	Pilot-grid ZF representation $H_{\text{in},k,t} = Y_{k,t}/X^{\text{pilot}}_{k,t}$, with real and imaginary components stacked	$[B, 2, 100, 4]$	No trainable layer	0
Shared real–imaginary embedding	Conv2d $2 \to 32$, kernel $(7, 1)$; Conv2d $32 \to 32$, kernel $(7, 1)$	$[B, 32, 88, 4]$	LeakyReLU(0.5) after each convolution	7,680
CFO feature MLP	Flatten 11264; Linear $11264 \to 512$; Linear $512 \to 256$; Linear $256 \to 128$	$[B, 128]$	Dropout(0.3), SiLU	5,931,904
CFO head	Tanh; Linear $128 \to 1$; Tanh	$[B, 1]$	Output constrained to $[-1, 1]$	129
Confidence gate	Tanh; Linear $128 \to 1$; Sigmoid	$[B, 1, 1]$	$\mathrm{g} = \sigma(a_{\mathrm{g}})$	129
CFO attention scale	SiLU; Linear $128 \to 32$; SiLU	$[B, 32]$	Multiplicative feature modulation	4,128
CFO attention bias	SiLU; Linear $128 \to 32$; SiLU	$[B, 32]$	Additive feature modulation	4,128
$\mathbf{H}_{\text{scale}}$ head	Flatten modulated feature; Linear $11264 \to 800$; reshape into real and imaginary channels	$[B, 2, 100, 4]$	Unconstrained linear output	9,012,000
$\mathbf{H}_{\text{prior}}$ head	Flatten modulated feature; Linear $11264 \to 800$; reshape into real and imaginary channels	$[B, 2, 100, 4]$	Unconstrained linear output	9,012,000
Fixed Gaussian interpolator	Normalised Conv2d with fixed frequency-axis Gaussian kernel, kernel size $(13, 1)$ and $\sigma = 3.0$; temporal dimension has size 1 (no temporal smoothing)	$[B, 2, 600, 4]$	No trainable parameters	0
Active estimator total	Modules called in the reported forward path	–	–	23,972,098

Implementation note. Pilot estimates are scattered to their active-subcarrier locations on a zero-filled dense grid, and the fixed Gaussian convolution is normalised by the convolved pilot mask.

Repeated MRI blocks are defined as follows. An EfficientConvBlock applies a bias-free depthwise 3×3 convolution, LayerNorm2d, SiLU, a pointwise 1×1 convolution, LayerNorm2d, and SiLU, with a residual connection when input and output shapes match. A squeeze-and-excitation block (SEBlock) applies global average pooling, bias-free Linear $C\to C/16$, ReLU, bias-free Linear $C/16\to C$, Sigmoid, and channel reweighting.

Table S6. PEIL architecture breakdown for MRI.

Component	Layers or operation	Output or role	Parameters
Estimator input	Zero-filled coil images obtained by inverse fast Fourier transform (FFT), arranged as $[B, N_c, 2, H, W]$ and reshaped to $[B, 2N_c, H, W]$ with $N_c = 15$	Input derived from acquired k-space	0
Stem	Conv2d $30\to48$, kernel 3×3; two EfficientConvBlocks $48\to48$	Shared feature map $[B, 48, H, W]$	18,960
Down1	Conv2d $48\to96$, kernel 3×3, stride 2; EfficientConvBlock $96\to96$; SEBlock(96)	Shared feature map $[B, 96, H/2, W/2]$	53,280
Down2	Conv2d $96\to192$, kernel 3×3, stride 2; EfficientConvBlock $192\to192$; SEBlock(192)	Shared feature map $[B, 192, H/4, W/4]$	210,240
ρ_{CG} head	Adaptive average pooling; Linear $192\to64$; Linear $64\to1$; Sigmoid, scaled by 1.0 and shifted by 0.01	Scalar Tikhonov weight used by the fixed CG solver	12,417
Sensitivity decoder	Bilinear upsampling with skip connections; Conv2d $288\to96$, kernel 1×1; EfficientConvBlock $96\to96$; SEBlock(96); Conv2d $144\to48$, kernel 1×1; EfficientConvBlock $48\to48$; SEBlock(48)	Coil-sensitivity feature map $[B, 48, H, W]$	49,680
Sensitivity shared head	EfficientConvBlock $48\to48$	Shared feature for sensitivity phase and amplitude heads	2,976
Sensitivity phase head	Conv2d $48\to48$, kernel 1×1; SiLU; EfficientConvBlock $48\to48$; Conv2d $48\to48$, kernel 3×3; Conv2d $48\to30$, kernel 1×1	Unit-magnitude complex phase for 15 coil maps, obtained by ϵ-stabilised complex normalisation	27,582
Sensitivity amplitude head	Conv2d $48\to48$, kernel 1×1; SiLU; EfficientConvBlock $48\to48$; Conv2d $48\to48$, kernel 3×3; Conv2d $48\to15$, kernel 1×1	Positive amplitude for 15 coil maps, produced by Sigmoid scaling and a small positive offset	26,847
Reference decoder	Bilinear upsampling with skip connections; Conv2d $288\to96$, kernel 1×1; EfficientConvBlock $96\to96$; SEBlock(96); Conv2d $144\to48$, kernel 1×1; EfficientConvBlock $48\to48$; SEBlock(48)	Image-domain reference feature map $[B, 48, H, W]$	49,680
Reference shared head	EfficientConvBlock $48\to48$	Shared feature for reference phase and amplitude heads	2,976
Reference phase head	Conv2d $48\to48$, kernel 1×1; SiLU; EfficientConvBlock $48\to48$; Conv2d $48\to48$, kernel 3×3; Conv2d $48\to2$, kernel 1×1	Unit-magnitude complex phase for $\mathbf{X}_{\mathrm{prior}}$, obtained by ϵ-stabilised complex normalisation	26,210
Reference amplitude head	Conv2d $48\to48$, kernel 1×1; SiLU; EfficientConvBlock $48\to48$; Conv2d $48\to48$, kernel 3×3; Conv2d $48\to1$, kernel 1×1	Positive amplitude for $\mathbf{X}_{\mathrm{prior}}$, produced by Sigmoid scaling and a small positive offset	26,161

Component	Layers or operation	Output or role	Parameters
Fixed CG solver	15 conjugate-gradient iterations using the multi-coil encoding operator and its adjoint	Final reconstructed image; no learnable update or post-solver correction	0
Shared encoder subtotal	Stem, Down1, and Down2	Shared features for all heads	282,480
ρ_{CG} subtotal	Scalar regularisation-weight head	Solver-conditioning weight	12,417
Coil-sensitivity branch subtotal	Sensitivity decoder, shared head, phase head, and amplitude head	Estimated coil sensitivity maps $\hat{\mathbf{S}}$	107,085
Image-domain reference branch subtotal	Reference decoder, shared head, phase head, and amplitude head	Solver-conditioning reference $\mathbf{X}_{\mathrm{prior}}$	105,027
Estimator total	All trainable PEIL estimator modules in MRI	Fixed CG solver excluded because it has no trainable parameters	507,009
